# Estrogen deficiency induces pelvic floor muscle atrophy via ERα/GLUT4 pathway

**DOI:** 10.1371/journal.pone.0349371

**Published:** 2026-05-14

**Authors:** Xiaoyu Huang, Mengqi Zhou, Ying Wang, Mao Chen, Ya Xiao, Lingyun Li, Fangyi Zhu, Liying Chen, Xiaoyu Tian, Shiman Wu, Bingshu Li, Li Hong

**Affiliations:** 1 Department of Gynecology and Obstetrics, Renmin Hospital of Wuhan University, Wuhan, China; 2 Pelvic Floor Research Centre of Hubei Province, Renmin Hospital of Wuhan University, Wuhan, China; Fujita Health University, JAPAN

## Abstract

Pelvic floor dysfunction (PFD) is a common disease in women that seriously affects physical and psychological health. Menopause-associated estrogen reduction is one of the risk factors. However, the role and mechanism of estrogen in PFD remains unclear. In this study, we observed atrophy of both fast and slow muscle fibers in the pelvic floor muscle (PFM) of ovariectomized rats, accompanied by decreased expression of estrogen receptor α (ERα). Estrogen deficiency severely impaired the proliferation, differentiation, and mitochondrial function of C2C12 myoblasts and increased apoptosis, which could be rescued by ERα agonist. Mechanistically, estrogen deficiency led to the downregulation of ERα, which in turn suppressed the expression of glucose transporter 4 (GLUT4) and its trafficking regulator Rac family small GTPase 1 (RAC1). This disruption abolished the critical co-localization of GLUT4 with RAC1, resulting in defective glucose uptake, mitochondrial dysfunction, and ultimately impaired myoblast proliferation and differentiation. Both ERα activation and GLUT4 overexpression rescued these defects. Thus, our study delineates a novel ERα/GLUT4 pathway that mediates PFM atrophy under estrogen deficiency conditions, providing a potential therapeutic target for PFD.

## 1. Introduction

Pelvic floor dysfunction (PFD) is a common disease in middle-aged and elderly women. More than 30% of female individuals have experienced PFD-related symptoms, such as urinary incontinence, pelvic organ prolapse, fecal incontinence and sexual dysfunction [1,2]. PFD occurs more frequently in women during pregnancy, postpartum and postmenopausal periods [3]. Severe PFD significantly affects the daily life of patients, bringing embarrassment and leading to social isolation [4]. Injury of pelvic floor muscle (PFM) is closely related to PFD susceptibility [5–8]. Pathological changes have been found in PFM in both PFD patients and animal model [9,10]. Due to the difficulty in obtaining human PFM samples, current studies on PFD mainly focus on pelvic floor ligaments, vaginal walls and other tissues. The role and mechanism of PFM in the occurrence and development of PFD are still unclear.

Epidemiological investigations have found that strength of female skeletal muscle is correlated with estrogen levels [11–13]. It has been reported that estrogen can rescue abnormal collagen deposition in the intermuscular tissue by remodeling the extracellular matrix and improve the prognosis of PFD patients [14]. The effect of estrogen depends not only on its concentration but also on the expression of estrogen receptor (ER). Altered ERα and ERβ ratio have been observed in patients with PFD [15,16]. Estrogen interventions on different tissues can lead to conflicting results, which may be associated with the difference in ER type and activity. However, the role of estrogen in PFM and its specific mechanisms remain to be further explored.

In this study, we employed ovariectomized (OVX) rats and estrogen‑deficient (EsD) C2C12 myoblasts to investigate the role of estrogen in PFM homeostasis. We found that estrogen loss attenuates ERα activity in the PFM, which in turn down‑regulates glucose transporter 4 (GLUT4) and disrupts its co‑localization with Rac family small GTPase 1 (RAC1). These alterations lead to abnormal glucose metabolism and mitochondrial injury in muscle cells, ultimately impairing cell proliferation and differentiation and resulting in PFM atrophy. Our findings thus establish the critical role of the estrogen‑regulated ERα/GLUT4 pathway in PFM atrophy under EsD conditions and provide a novel theoretical basis for PFD treatment.

## 2. Materials and methods

### 2.1. Materials

Phenol red-free cell culture medium Dulbecco’s Modified Eagle Medium (DMEM) was from Life Technologies Corporation (Carlsbad, CA 92008, USA) (Cat No. 31053036, lot No. 2193168). Quality Biological Inc provided the fetal bovine serum, while Thermo-Fisher Scientific supplied 100X Penicillin/Streptomycin solution, 100X glutamine solution, and 100X trypsin/EDTA solution of top-notch quality. 17β-estradiol (> 98%) was purchased from Sigma-Aldrich (St. Louis, USA). Propyl pyrazole triol (PPT, Cat. No. HY-100689) and AZD9496 (Cat. No. HY-12870) were acquired from MCE (New Jersey, USA). Lentiviral vectors (NM_009204-EGFP) with over-expression of GLUT4 (46721−2) were created by Shanghai Genechem Co. Ltd. These vectors were infected according to the manufacturer’s protocol.

### 2.2. Cell culture and treatment

C2C12 cells were purchased from Cobioer Biosciences CO., LTD (Nanjing, China) and cultured in growth medium (DMEM supplemented with 10% fetal bovine serum and 1% penicillin-streptomycin) and incubated at 37 °C with 5% CO_2_. Upon reaching 80–90% confluence, C2C12 cells were induced to differentiate by switching to a differentiation medium (DMEM supplemented with 2% horse serum and 1% penicillin-streptomycin). To establish an estrogen-deficient (EsD) model, cells were cultured in phenol red-free DMEM. Within this model, the ERα agonist and 17β-estradiol treatment groups were exposed to 100 nM of the respective compound. In contrast, for the ERα inhibition group, C2C12 cells maintained under normal culture conditions were treated with 100 nM ERα inhibitor.

### 2.3. Animals and treatment

All animal experiments were conducted in accordance with protocols approved by the Ethics Committee for Animal Experimentation of Renmin Hospital of Wuhan University (WDRM20200805). Female Sprague-Dawley rats (10 weeks old) were housed under controlled conditions (20 ± 2°C, 12-h light/dark cycle) with free access to food and water throughout the study. Rats were OVX under isoflurane anesthesia (4% for induction, 2% for maintenance) to induce estrogen deficiency, with sham-operated (Sham) rats serving as controls. For *in vivo* ERα intervention, OVX rats received the ERα agonist PPT (100 μg/kg/day, intraperitoneally), whereas Sham rats received the ERα inhibitor AZD9496 (60 μg/kg/day, intraperitoneally) for 12 weeks. Each group consisted of 6 rats. At the end of the experiment, all rats were euthanized by cervical dislocation under deep isoflurane anesthesia to minimize suffering, and the PFM were harvested as previously described [9]. Animal health and behavior were monitored daily, and no unexpected mortality or morbidity occurred during the study.

### 2.4. Bioinformatic analysis

We obtained datasets with human biological background and samples clearly labeled with age and sex information from GEO database: expression profiling by high throughput sequencing GSE164471 and GSE129643 and array dataset GSE8479 [17–19]. To reduce the heterogeneity among tissues, we selected only biological samples from the lateral femoral muscle. R (version 4.3.0) and BiocManager software packages were utilized for preprocessing. To meet the needs of experimental design, we selected women of childbearing age between 20 and 30 years old as the non-menopausal group samples from the biological samples, and the samples of elderly women aged 60 years old or above were categorized as the postmenopausal group samples. FunRich 3.1.3 analyzed the expression of pertinent genes by representing trends in a heatmap. To remove the varying batch experimental influences among GSE164471 and GSE129643, the R package surrogate variable analysis [20], was used to combine the expression matrix. We also extracted the expression of *SLC2A4* in other organs for comparison in GSE1839 and GSE2251 [21,22], with samples affected by estrogen levels selected.

### 2.5. Histology and morphometric analysis

PFM were extracted, fixed in the muscle fixation solution (Servicebio, Wuhan, China), and then encased in paraffin. For evaluating tissue structure, we used hematoxylin and eosin (H&E) and MASSON staining on cross-sections. The microscope (Olympus, Tokyo, Japan) was used to examine the sections.

### 2.6. Immunofluorescence staining

For staining of PFM, we collected the samples frozen by liquid nitrogen-cooled isopentane in Tissue-Tek OCT (SAKURA, Japan) and then sliced muscles into 5-μm sections by a cryostat (CM1850, Leica, Germany). The sections were air-dried for 5 minutes, then treated with cold acetone for 10 minutes at 4 degrees Celsius, rinsed with PBS, and then blocked with 10% goat serum (Beyotime, Shanghai, China) in PBST for 1 hour at room temperature. Following the blocking process, the sections were left to incubate overnight at 4 °C with primary antibodies against MyHC-fast (GB112130, 1:500, Servicebio), MyHC-slow (GB121857, 1:500, Servicebio), ERα (GB111843−100, 1:200, Servicebio) and ERβ (GB115454−100, 1:200, Servicebio). The following day, the segments were rinsed thrice with PBST and exposed to corresponding fluorescent secondary antibodies at room temperature for 1 hour. Subsequently, the segments were affixed with aqueous mounting solution (with DAPI) (Thermo Fisher Scientific, USA) and captured using a fluorescence microscope (Olympus).

### 2.7. EdU assay

Cell proliferation was assessed using the EdU assay. Following treatments, cells were incubated with 10 μM EdU in serum-free DMEM for 2 hours. Cells were then washed with PBS, fixed with 4% formaldehyde for 15 minutes, and permeabilized with 0.5% Triton X-100 for 10 minutes. Following PBS wash, the nuclei were stained with Hoechst33342. The presence of EdU was identified through a fluorescent-azide coupling reaction in the organization. Following application of anti-fluorescence quenching mounting media (G1401, Servicebio), images were captured with a fluorescence microscope (Olympus) utilizing cellSens standard software.

### 2.8. Terminal deoxynucleotidyl transferase dUTP nick-end labeling (TUNEL) assay

Apoptosis was assessed using a TUNEL assay. Following treatment, cells were fixed with 4% paraformaldehyde at room temperature for 1 hour, permeabilized with 0.1% Triton X-100 for 10 minutes, and washed with PBS. Subsequent staining was performed using a commercial TUNEL kit (Roche, Switzerland) according to the manufacturer’s protocol. TUNEL-positive cells were visualized and counted under an inverted fluorescence microscope (Olympus).

### 2.9. Total RNA extraction and quantitative reverse-transcription polymerase chain reaction (qRT-PCR)

Total RNA was extracted using TRIzol reagent (Takara, Otsu, Japan) following the manufacturer’s protocol and subsequently reverse-transcribed into cDNA using the Hifair II 1st Strand cDNA Synthesis Kit (YEASEN, Shanghai, China). Real-time PCR analysis was performed with Hieff qPCR SYBR Green Master Mix (YEASEN) using the CFX96 Real-time PCR detection system (Bio-Rad, California, USA). Gene expression levels were calculated using the 2^-ΔΔCT^ method with *Actb* as the internal reference, and data were normalized to the control group. The primer sequences used are listed in Table 1.

**Table 1:** The primer sequences for qRT-PCR.

Gene	Primer Sequence (5’-3’)
***Esr1***	F-CCTCCCGCCTTCTACAGGT R-TCAGGCAGGTCAAAGGAACT
***Esr2***	F-CTGGTCTGGGTGATTTCG R-ACTGATGTGCCTGACATGAGAAAG
***Myh7***	F-GTGGCTCCGAGAAAGGAAG R-GAGCCTTGGATTCTCAAACG
***Myh2***	F-CAGAGGCAAGTAGTGGTGGA R-CAAATTCTCTCTGAACAGGGCA
***Ppargc1a***	F-AGACGGATTGCCCTCATTTGA R-TGTAGCTGAGCTGAGTGTTGG
***Slc2a4***	F-TATTTGGCTTTGTGGCCTTC R-CGGCAAATAGAAGGAAGACG
***Rac1***	F-CCTCCCGCCTTCTACAGGT R-TCAGGCAGGTCAAAGGAACT
***Actb***	F-CTACCTCATGAAGATCCTGAC R-CACAGCTTCTCTTTGATGTCAC

### 2.10. Western blotting

Total proteins were extracted from cells and tissues using RIPA Lysis Buffer (Beyotime) supplemented with protease and phosphatase inhibitors (MCE), and protein concentrations were determined with a BCA Assay Kit (Beyotime). After separation by SDS-PAGE, proteins were transferred to PVDF membranes and incubated overnight at 4°C with primary antibodies. The following primary antibodies were used: GLUT4 (66846–1-Ig, 1:2000, Proteintech, Wuhan, China), RAC1 (66122–1-Ig, 1:3000, Proteintech), TFAM (22586–1-AP, 1:5000, Proteintech), PGC1α (66369–1-Ig, 1:5000, Proteintech), β-actin (66009–1-Ig, 1:20000, Proteintech), MyHC (ab37484, 1:1000, Abcam, Cambridge, UK), ERα (GB111843–100, 1:1000, Servicebio) and ERβ (GB115454–100, 1:1000, Servicebio). Subsequently, membranes were washed with TBST, incubated with goat anti-mouse HRP-conjugated secondary antibody (SA00001–1, 1:5000, Proteintech) or goat anti-rabbit HRP-conjugated secondary antibody (SA00001–2, 1:5000, Proteintech) for 1 hour at room temperature, and visualized using a Bio-Rad ChemiDoc XRS + Gel Imaging System. Band intensities were quantified with ImageJ software and normalized to the corresponding β‑actin loading control. The normalized values were then expressed as fold change relative to the control group.

### 2.11. JC-1 staining

Mitochondrial membrane potential was assessed using the JC-1 assay kit. Briefly, cells were washed with PBS and incubated with JC-1 dye (diluted 1:1 in DMEM) for 30 minutes at 37 °C. After washing with the kit’s staining buffer, images were captured using an optical microscope. Quantitative analysis was performed by flow cytometry (FACS Calibur, BD Biosciences), and data were analyzed with FlowJo software.

### 2.12. Measurement of intracellular mitochondria mass

Mitochondrial mass was assessed using MitoTracker Red CMXRos (C1049B, Beyotime). Briefly, cells were incubated with the dye at 37 °C for 30 min, followed by flow cytometric analysis on a BD FACS Calibur system. Data were processed using FlowJo software.

### 2.13. Cell cycle experiment

Cell cycle distribution was analyzed using a commercial kit (C1052, Beyotime). Cells were harvested, fixed in 70% ethanol at 4°C for 2 hours, and then stained in the dark for 30 minutes with a solution containing propidium iodide (0.05 mg/mL), RNase A (1 mg/mL), and 0.3% Triton X-100. DNA content (PI intensity) was measured by flow cytometry (Beckman Coulter, USA), and the proportions of cells in G1, S, and G2/M phases were quantified using FlowJo software.

### 2.14. Glucose uptake assay

Glucose uptake was assessed using the fluorescent glucose analog 2-NBDG. A 100 μM working solution was prepared by dissolving 1 mg of 2-NBDG powder in 100 μL of DMSO. After washing with PBS, harvested cells were incubated with 500 μL of the 2-NBDG solution for 15 minutes. Cellular fluorescence intensity was then measured by BD FACS Calibur flow cytometry system and analyzed with FlowJo software.

### 2.15. Statistical analysis

Data were expressed as the mean ± standard deviation. Statistical analyses were conducted to compare multiple groups using one-way ANOVA, followed by a Tukey post-hoc test. A two-tailed Student t-test was conducted to compare two conditions. p < 0.05 was considered significant. Statistical analysis was conducted using GraphPad Prism software (San Diego, California, USA). ScatterJ, a plug-in for analyzing colocalization in ImageJ software, was used to compute Pearson’s and Manders’ coefficients.

## 3. Results

### 3.1. Estrogen deficiency induces PFM atrophy in OVX rats

**Fig 1:**
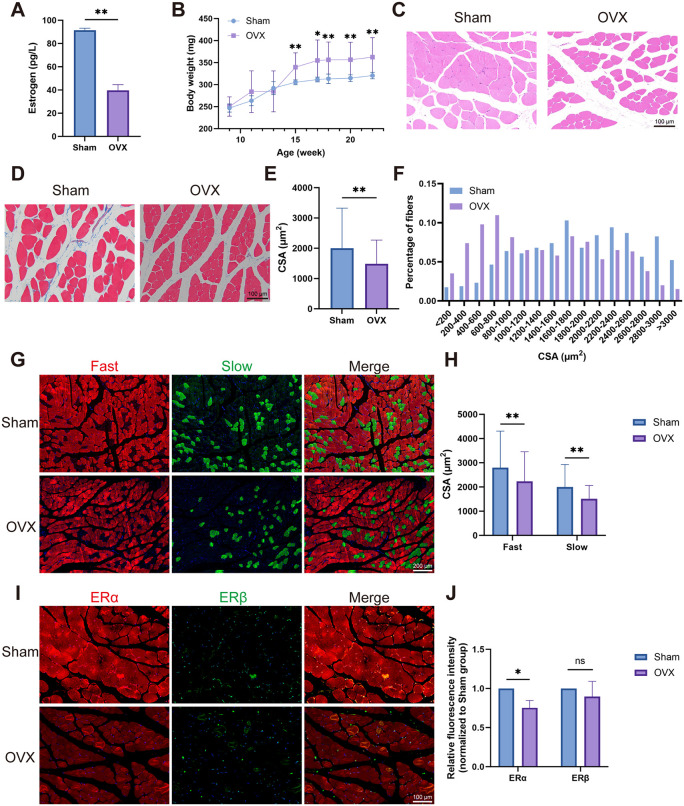
Estrogen deficiency induces PFM atrophy associated with ERα downregulation in OVX rats. **(A)** Serum estrogen levels at 12 weeks after surgery. **(B)** Body weight monitoring from 5 to 12 weeks after surgery. **(C)** H&E staining results of the PFM of rats in the OVX and Sham groups. **(D, E)** MASSON staining results of the PFM of rats in the OVX and Sham groups. **(F)** Distribution of myofiber CSA in the PFM of rats in the OVX and Sham groups. **(G, H)** Fast (red) and slow (green) muscle staining results of the PFM of rats in the OVX and Sham groups. **(I, J)** Immunofluorescence results for ERα (red) and ERβ (green) of the PFM of rats in the OVX and Sham groups. Data are presented as mean ± SD. *p < 0.05, **p < 0.01, ns, no significance.

First, a rat model of estrogen deficiency was constructed by OVX surgery and the characterization of the PFM was assessed. Serum estrogen concentration in OVX rats decreased significantly 12 weeks after operation (Fig 1A), confirming successful model establishment. Body weight of OVX rats was always significantly higher than that of Sham rats at 5–12 weeks (15–22 weeks of age) after surgery (Fig 1B), suggesting systemic metabolic effects of estrogen deficiency. H&E and MASSON stains showed that the mean cross-sectional area (CSA) of PFM significantly decreased in OVX rats (Fig 1C**-**1F), indicating muscle atrophy. Both fast and slow fibers were present in the PFM, and notably, the CSA of both fiber types was significantly reduced in OVX rats compared to the Sham group (Fig 1G, 1H). In addition, both ERα and ERβ expression were detected in PFM (Fig 1I, 1J). Immunofluorescence analysis revealed that ERβ localized primarily to myonuclei and intermuscular tissues, whereas ERα was present in both myonuclei and myofibers. 12 weeks after the surgery, the ERα expression was significantly downregulated in OVX rats, while the ERβ expression was not significantly altered. Together, these findings indicate that ERα is the primary estrogen receptor in PFM, and that estrogen deficiency reduces its expression, leading to atrophy of both slow and fast muscle fibers.

### 3.2. Estrogen deficiency impairs myoblast function and mitochondrial integrity

**Fig 2:**
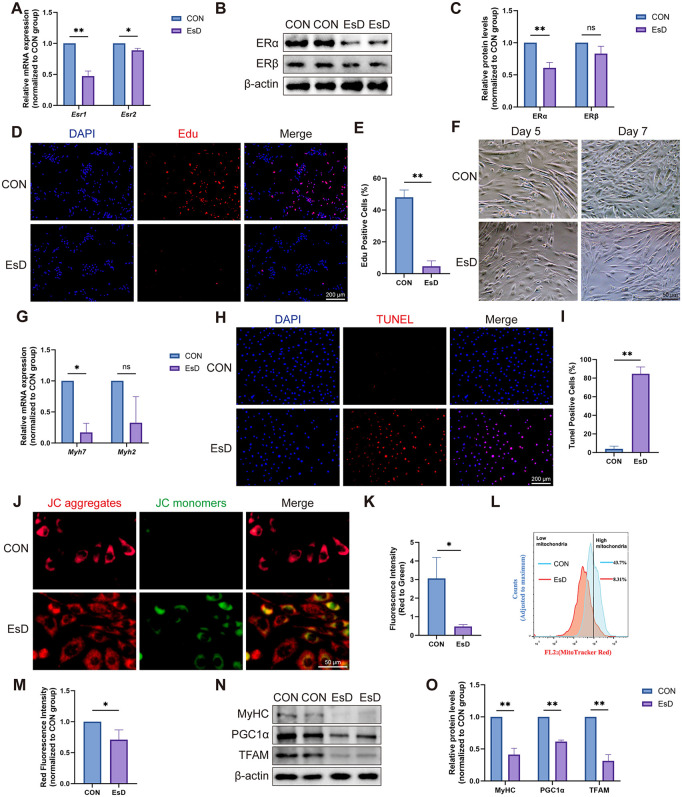
Estrogen deficiency impairs C2C12 myoblast function and mitochondrial integrity. **(A)** qRT-PCR results of *Esr1* and *Esr2* in C2C12 cells under EsD conditions. **(B-C)** Western blotting results of ERα and ERβ in C2C12 cells under EsD conditions. **(D, E)** EdU results in C2C12 cells under EsD conditions. **(F)** C2C12 cells differentiated into myotubes on Day 5 and Day 7. **(G)** qRT-PCR results of *Myh7* and *Myh2* in C2C12 cells under EsD conditions. **(H,** **I)** TUNEL results in C2C12 cells under EsD conditions. **(J, K)** Results of JC-1 assay in C2C12 cells under EsD conditions. **(L,M)** Flow sorting results for mitochondrial number in C2C12 cells under EsD conditions. **(N,O)** Western blotting results of MyHC, PGC1α and TFAM in C2C12 cells under EsD conditions. Data are presented as mean ± SD. *p < 0.05, **p < 0.01, ns, no significance.

Given the role of myoblasts in muscle repair and maintenance [23], we investigated the impact of estrogen deficiency on C2C12 myoblasts. Both qRT-PCR and Western blotting analyses confirmed significant downregulation of ERα under estrogen-deficient (EsD) conditions (Fig 2A-2C), consistent with *in vivo* findings, while ERβ showed little change, reinforcing ERα as the primary mediator. Estrogen deficiency markedly impaired the proliferation (Fig 2D, 2E) and differentiation (Fig 2F) of C2C12 cells. This was accompanied by a specific decrease in the mRNA expression of the slow-fiber marker *Myh7*, but not the fast-fiber marker *Myh2* (Fig 2G). Furthermore, TUNEL assay revealed a significant increase in apoptosis in the EsD group (Fig 2H, 2I).

Previous studies have found that estrogen protects cellular and mitochondrial functions [24–26]. Consistently, our results found an impaired mitochondrial membrane potential (Fig 2J, 2K) and a reduction in the number of mitochondria (Fig 2L, 2M) in the EsD group. Furthermore, not only MyHC (a differentiation marker) but also mitochondrial biogenesis-related factors TFAM and PGC1α were down-regulated in the EsD group (Fig 2N, 2O). Collectively, these data demonstrate that estrogen deficiency induces C2C12 cell damage, myotube atrophy, and mitochondrial dysfunction, with mitochondrial damage likely preceding or directly contributing to the differentiation defect.

### 3.3. ERα activation restores myoblast health and mitochondrial function impaired by estrogen deficiency

**Fig 3:**
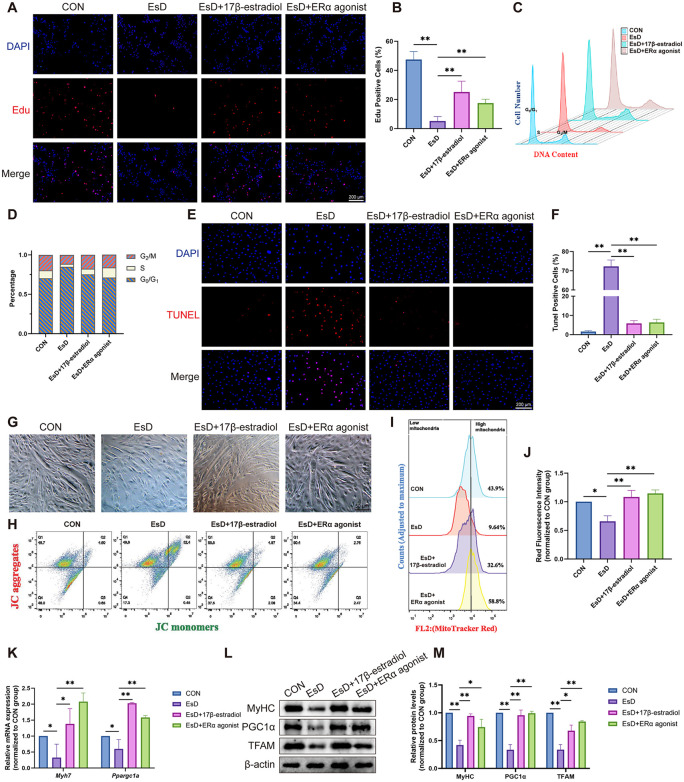
Effects of ERα agonist in estrogen-deficient C2C12 cells. **(A, B)** EdU results in C2C12 cells under different culture conditions. **(C-D)** Cell cycle experiment results in C2C12 cells under different culture conditions. **(E, F)** TUNEL results in C2C12 cells under different culture conditions. **(G)** C2C12 cells differentiated into myotubes on Day 7. **(H)** Mitochondrial membrane potential levels of C2C12 cells under different culture conditions. **(I, J)** Mitochondrial quantity of C2C12 cells under different culture conditions. **(K)** qRT-PCR results of *Myh7* and *Ppargc1a* in C2C12 cells under different culture conditions. **(L, M)** Western blotting results of MyHC, PGC1α and TFAM in C2C12 cells under different culture conditions. Data are presented as mean ± SD. *p < 0.05, **p < 0.01, ns, no significance.

Given the specific reduction of ERα in the PFM of OVX rats and in the EsD C2C12 cells, we next investigated whether ERα is the primary mediator of estrogen’s protective effects using an ERα agonist. The results showed that the ERα agonist rescued the estrogen deficiency-induced impairments in C2C12 cells, including decreased proliferation, cell cycle arrest, increased apoptosis, and impaired differentiation, with effects that paralleled the trend observed with 17β-estradiol treatment (Fig 3A-3G). Furthermore, the agonist restored the diminished mitochondrial membrane potential and mitochondrial number (Fig 3H-3J), and reversed the downregulation of mitochondrial biogenesis factors (TFAM and PGC1α) as well as myogenic markers (*Myh7* and MyHC) (Fig 3K-3M). These findings not only confirm that ERα mediates the protective effects of estrogen on C2C12 myoblasts but also directly link ERα to mitochondrial function.

### 3.4. GLUT4 is involved in the muscle atrophy caused by estrogen deficiency

**Fig 4:**
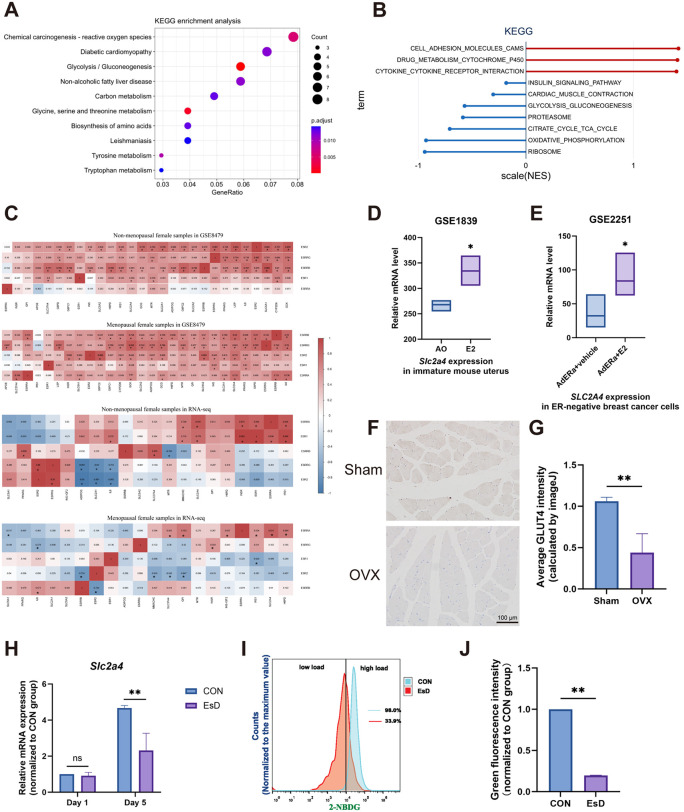
Identification and validation of GLUT4 as a key estrogen target in skeletal muscle metabolism. **(A)** KEGG pathway enrichment analysis of DEGs in skeletal muscle transcriptomes from pre- versus post-menopausal women. **(B)** GSEA of KEGG pathways. Red and blue indicate overall up- or down-regulation in post-menopausal samples, respectively. NES, normalized enrichment score. **(C)** Correlation heatmap between estrogen receptor genes and glucose metabolism-related genes. Color and number in each cell represent the Pearson correlation coefficient. **(D)** Expression of *Slc2a4* in the uterus of immature mice treated with estrogen. **(E)** Effect of estrogen on *SLC2A4* expression in ER-negative breast cancer cells. **(F, G)** Immunohistochemical staining of GLUT4 in the PFM of Sham and OVX rats. **(H)** qRT-PCR results of *Slc2a4* during C2C12 differentiation. **(I, J)** Glucose uptake assay in C2C12 cells on Day 3 of differentiation. Data are presented as mean ± SD. *p < 0.05, **p < 0.01, ns, no significance.

To identify potential targets, we performed bioinformatic analysis on skeletal muscle genomic data from pre- and post-menopausal women (GSE164471 and GSE129643), groups with markedly different estrogen levels. Kyoto Encyclopedia of Genes and Genomes (KEGG) and Gene Set Enrichment Analysis (GSEA) revealed that the differential expressed genes (DEGs) were significantly associated with glucose metabolism pathways (Fig 4A, B). Given the close interplay between glucose metabolism and mitochondrial function [27], and our observation of mitochondrial damage in EsD cells, we hypothesized that estrogen regulates skeletal muscle partly through glucose metabolism. To explore this, we analyzed the correlation between estrogen receptors and glucose metabolism genes across the gene chip dataset GSE8479, and RNA-seq datasets GSE164471 and GSE129643. A significant correlation between *ESR1* (encoding ERα) and *SLC2A4* (encoding GLUT4) expression was found in pre-menopausal but not post-menopausal samples (Fig 4C). Furthermore, *SLC2A4* expression in other estrogen-sensitive tissues showed a consistent positive association with estrogen levels (Fig 4D, E). These bioinformatic predictions identified *SLC2A4* as a key potential target of estrogen in skeletal muscle metabolic regulation.

Building on the bioinformatic prediction of *SLC2A4* as a key target, we next validated its role in experimental models. *In vivo* validation showed that GLUT4 protein was significantly downregulated in the PFM of OVX rats (Fig 4F, 4G). During C2C12 myoblast differentiation, while *Slc2a4* mRNA expression increased over time in both groups, its level was consistently and significantly lower under EsD conditions (Fig 4H), confirming estrogen-dependent regulation. Consequently, glucose uptake was impaired in C2C12 cells under EsD conditions (Fig 4I, 4J). In summary, these findings confirm the important role of GLUT4 in regulating PFM atrophy associated with estrogen deficiency.

### 3.5. ERα maintains myoblast function and mitochondrial integrity through GLUT4

**Fig 5:**
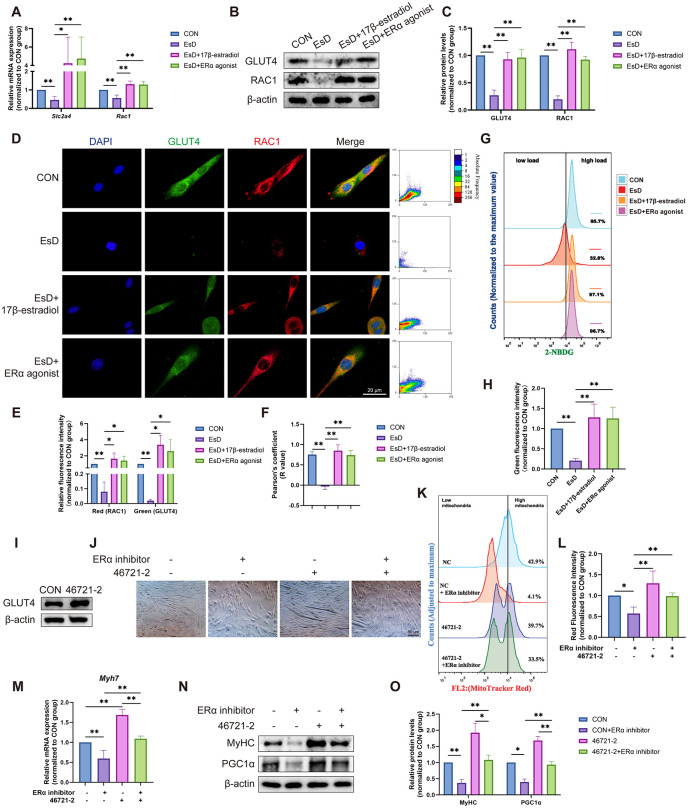
ERα maintains myoblast function and mitochondrial integrity through GLUT4. **(A)** qRT-PCR results of *Slc2a4* and *Rac1* in C2C12 cells under different culture conditions. **(B, C)** Western blotting results of GLUT4 and RAC1 in C2C12 cells under different culture conditions. **(D-F)** Immunofluorescence staining and co-localization analysis of GLUT4 (green) and RAC1 (red) in C2C12 cells under different culture conditions. Nuclei are counterstained with DAPI (blue). **(G, H)** Glucose uptake assay in C2C12 cells under different culture conditions. **(I)** Establishment of a GLUT4-overexpressing C2C12 cell line. **(J)** Myotube images of C2C12 cells under different conditions on Day 7 of differentiation. **(K, L)** Mitochondrial quantity of C2C12 cells under different culture conditions. **(M)** qRT-PCR results of *Myh7* in C2C12 cells under different culture conditions. **(N, O)** Western blotting results of MyHC and PGC1α in C2C12 cells under different culture conditions. Data are presented as mean ± SD. *p < 0.05, **p < 0.01.

Having established that ERα mediates the beneficial effects of estrogen and that GLUT4-regulated glucose metabolism contributes to PFM atrophy under estrogen deficiency, we next investigated whether ERα acts through GLUT4 in C2C12 cells. Activation of ERα or supplementation with 17β-estradiol in EsD cells upregulated both mRNA and protein expression of GLUT4 (Fig 5A**-**5C). Since RAC1 is essential for GLUT4 vesicle translocation to the membrane [28,29], we also assessed its expression. Similarly to GLUT4, RAC1 was downregulated under EsD conditions, and this down-regulation was reversed by ERα activation or 17β-estradiol supplementation (Fig 5A**-**5C). Importantly, their subcellular localization was also affected by estrogen deficiency: the significant co-localization observed in control cells was abolished in EsD cells and restored upon ERα activation or 17β-estradiol treatment (Fig 5D**-**5F). Accordingly, the impaired glucose uptake capacity in EsD cells was rescued by activating ERα or supplementing with 17β-estradiol, as measured by 2-NBDG uptake assay (Fig 5G, 5H). Thus, ERα activation restores the disrupted trafficking of GLUT4/RAC1 and the resulting glucose uptake deficit caused by low estrogen.

To further establish the central role of GLUT4, we successfully generated a C2C12 cell line stably overexpressing GLUT4 (46721−2) (Fig 5I). Strikingly, GLUT4 overexpression rescued the impaired C2C12 myoblast differentiation (Fig 5J) and the reduction in mitochondrial number (Fig 5K, 5L) induced by estrogen deficiency or ERα inhibition. Besides, GLUT4 overexpression also partially restored the expression levels of myogenic markers *Myh7* and MyHC, as well as the mitochondrial regulator PGC1α (Fig 5M**-**5O). These results demonstrate that GLUT4 is a critical downstream effector of ERα signaling in maintaining myoblast function and mitochondrial integrity.

### 3.6. *In vivo* experiments validated the effect of ERα/GLUT4 pathway on PFM

**Fig 6:**
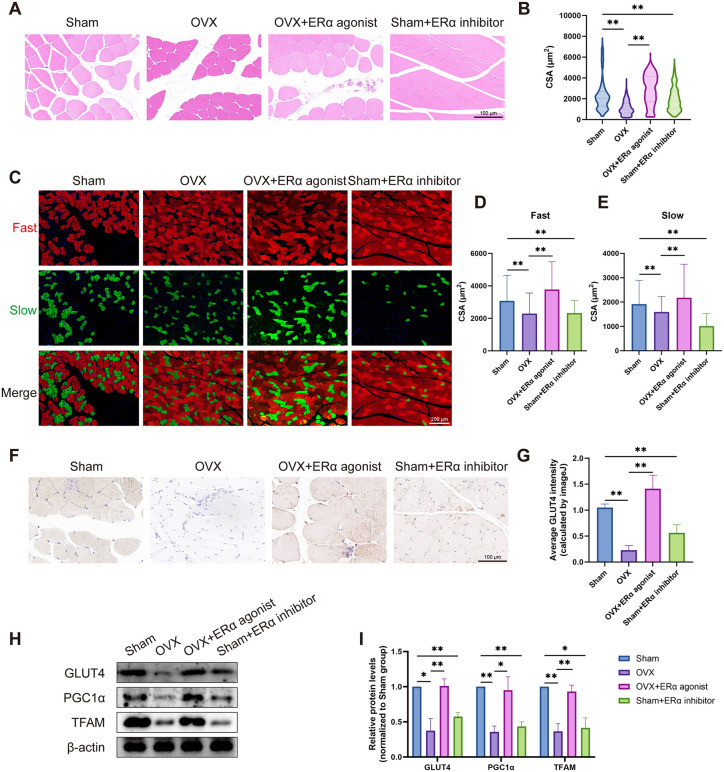
*In vivo* experiments to validate the effect of ERα/GLUT4 axis on PFM. **(A, B)** H&E staining results of the PFM of rats in different groups. **(C-E)** Fast (red) and slow (green) muscle staining results of the PFM of rats in different groups. **(F, G)** Immunohistochemical staining of GLUT4 in the PFM of rats in different groups. **(H,** **I)** Western blotting results of GLUT4, PGC1α and TFAM in the PFM of rats in different groups. Data are presented as mean ± SD. *p < 0.05, **p < 0.01.

To validate the role of the ERα/GLUT4 axis *in vivo*, we examined PFM morphology and molecular alterations in rats following 12-week interventions targeting ERα activity. Histological analysis revealed that PFM from OVX and ERα-inhibited groups contained a greater number of smaller, atrophic myofibers. ERα activation significantly increased the mean CSA of myofibers compared to the OVX group, whereas ERα inhibition significantly reduced it compared to the Sham group (Fig 6A, 6B). This pattern was consistent for both fast and slow muscle fiber types (Fig 6C-6E). At the molecular level, the expression of GLUT4, as determined by immunohistochemistry and Western blotting, was positively regulated by ERα activity, showing higher levels in the ERα-activated group and lower levels in the ERα-inhibited group compared to their respective controls (Fig 6F-6I). Similarly, the expression of the mitochondrial biogenesis markers PGC1α and TFAM was upregulated by ERα activation and downregulated by its inhibition (Fig 6H, 6I). These *in vivo* results are highly consistent with the *in vitro* findings, collectively confirming that ERα activity regulates GLUT4 expression in PFM, thereby influencing downstream metabolic and mitochondrial pathways and ultimately determining myofiber maintenance or atrophy.

## 4. Discussion

The occurrence and progression of PFD are closely associated with PFM. Various factors such as nutrition, hormones, and mechanical stimulation can lead to changes in muscle quality and function [30–32]. A randomized controlled trial also found that female participants showed a significant increase in muscle tissue CSA after 12 months of hormone replacement therapy compared to the control group [33], suggesting that estrogen plays a positive role in maintaining skeletal muscle quality. Using established estrogen-deficient animal and C2C12 myoblast models, this study explored the role of estrogen and its underlying molecular mechanisms in regulating muscle quality and function *in vivo* and *in vitro*.

PFM is skeletal muscle tissue composed of slow and fast muscle fibers. Slow muscle fibers can maintain tension for extended periods without fatigue, primarily serving to support the long-term stability of pelvic organs, while fast muscle fibers contract rapidly, providing force to maintain urethral pressure during rapid changes in abdominal pressure [34]. This study confirmed that ovariectomy lead to significant atrophy of both fast and slow fibers of PFM. Estrogen significantly impacts mitochondrial structure and energy metabolism pathways in female skeletal muscle, the deficiency of which led to the downregulation of PGC1α and TFAM. In C2C12 cell differentiation assay, we observed a significant down-regulation of *MYH7* (associated with slow muscle) due to decreased estrogen level, while *MYH2* (associated with fast muscle) showed no significant change. This further corroborated that the slow muscle fibers in PFM is more influenced by estrogen.

Estrogen exerts its physiological effects through ERs with distinct actions, including the classical nuclear receptors ERα and ERβ, as well as the membrane-associated G protein-coupled estrogen receptor (GPER) [16,35]. Therefore, identifying the predominant type of ER in female PFM is essential for investigating the specific mechanisms of estrogen’s effects on PFM quality. However, existing studies on ER expression in PFD have yielded inconsistent results. For instance, some studies have reported elevated ERβ protein in certain pelvic floor tissues of premenopausal patients [36], whereas others have found increased ERα expression and ERα/ERβ ratio in postmenopausal women [15], or upregulation of GPER and androgen receptor in uterosacral ligaments [16]. These discrepancies may arise from differences in tissue types (e.g., vaginal wall, uterosacral ligament, levator ani muscle), disease entities, menopausal status, and detection methods (protein versus mRNA).

In this context, our study confirmed that ERα is expressed at higher levels and more concentrated than ERβ in muscle cells within rat PFM. Moreover, ERα expression was significantly downregulated in PFM of OVX rats, whereas ERβ levels remained essentially unchanged, indicating that ERα represents the more critical ER subtype mediating estrogenic effects on PFM homeostasis. However, we acknowledge that estrogen may also signal through ERβ or GPER in a context‑dependent manner. Although ERβ expression remained unchanged in the PFM of our OVX rat model, we cannot entirely rule out its potential contribution—or that of GPER—in other pelvic floor tissues (e.g., ligaments or vaginal wall) or at different stages of estrogen deficiency. Nonetheless, the comparable efficacy of the ERα‑selective agonist and 17β‑estradiol in recapitulating the observed phenotypes, coupled with the lack of ERβ alteration in our model, argues strongly against a major role for ERα‑independent pathways in maintaining PFM myofiber homeostasis under our experimental conditions.

Previous studies have identified ERα as an important target for protecting skeletal muscle metabolic health [37]. Glucose metabolism plays crucial role in cellular energy production and conversion. Impaired glucose metabolism in skeletal muscle often leads to abnormalities in muscle quality, consequently resulting in metabolic diseases such as insulin resistance and diabetes [38–41]. We utilized both *in vivo* and *in vitro* experiments to confirm that ERα activity mediated the regulatory effects of estrogen on myoblast viability, differentiation capability, mitochondrial function, glucose metabolic homeostasis, and PFM quality.

Through bioinformatic analysis, this study reveals a strong correlation between menopausal status and glucose metabolism in skeletal muscle. Laakkonen et al. noted that menopausal status significantly affects energy metabolism pathways, including the citric acid cycle and oxidative phosphorylation [42]. Ronkainen et al. found that hormone replacement therapy alters the energy metabolism related gene pathways in the skeletal muscle of postmenopausal women [43]. Further analyses revealed a positive correlation between *SLC2A4* and *ESR1* in the skeletal muscle of premenopausal women, and *SLC2A4* expression was also positively associated with estrogen levels in other tissues. Given that GLUT4 is the principal glucose transporter in skeletal muscle and its dysfunction is tightly linked to metabolic dysregulation, we focused on its relationship with ERα signaling. This relationship has been inconsistently reported: while some studies suggest that ERα activation upregulates GLUT4 expression [44–46], others report no significant change in GLUT4 levels in ERα-knockout models [47]. Our findings help clarify this controversy by demonstrating that estrogen deficiency consistently downregulates GLUT4 in both rat PFM and C2C12 myoblasts, and that this downregulation is accompanied by measurable glucose metabolic disorder.

Although our study establishes the functional importance of the ERα/GLUT4 pathway in PFM atrophy under estrogen-deficient conditions, the precise molecular mechanism by which ERα regulates GLUT4 expression warrants further investigation. Notably, substantial evidence supports multiple indirect mechanisms by which ERα regulates GLUT4. First, ERα can upregulate GLUT4 expression by cooperating with transcription factor SP1, as demonstrated in adipocytes where ESR1 activation increases nuclear SP1 content, promotes SP1/ESR1 interaction, and enhances SP1 binding to the *Slc2a4* promoter [48]. Second, ERα can relieve NF-κB-mediated transcriptional repression of *Slc2a4*: the ESR1 agonist PPT reduces NF-κB binding activity to the *Slc2a4* promoter by approximately 50% [49]. Third, ERα can affect GLUT4 function via non-genomic pathways. For instance, 17β-estradiol induces SRC-mediated nucleusto-plasma membrane shuttling of ESR1, activates AKT phosphorylation, and promotes GLUT4 translocation and glucose uptake [50]; additionally, the ERα agonist resveratrol upregulates CAV-3 expression to facilitate GLUT4 translocation [51]. The intracellular trafficking of GLUT4 to the plasma membrane is mechanistically dependent on RAC1 [28,29]. Our study further demonstrates that estrogen deficiency severely disrupts GLUT4/RAC1 co-localization in C2C12 myoblasts, providing a mechanistic explanation for the observed deficit in glucose uptake.

Our data show that GLUT4 overexpression restores mitochondrial number and PGC1α expression in EsD myoblasts. However, it remains unclear whether this rescue is directly mediated by increased glucose uptake or involves additional signaling functions of GLUT4. The most straightforward mechanism is that enhanced glucose uptake fuels mitochondrial oxidative metabolism, elevating ATP and NADH levels, which activates AMPK and SIRT1 to induce PGC1α-dependent mitochondrial biogenesis [52–54]. This metabolic feedback is well-documented in skeletal muscle. Alternatively, GLUT4 has been implicated in non-metabolic signaling, such as modulating insulin receptor signaling [55] or regulating antiviral immunity [56], but direct evidence for such a role in myoblast mitochondrial regulation is lacking. To dissect these possibilities, future studies using non-metabolizable glucose analogs (e.g., 3-O-methylglucose) or glycolysis inhibitors (e.g., 2-deoxyglucose) could determine whether glucose transport per se, independent of its metabolism, is sufficient for mitochondrial rescue. Nonetheless, our current findings establish that GLUT4 restoration is sufficient to reverse the metabolic and mitochondrial deficits caused by estrogen deficiency, and we favor the interpretation that increased glucose uptake is the primary driver.

It is worth noting that OVX not only causes estrogen deficiency but also alters circulating levels of follicle-stimulating hormone (FSH), progesterone, and androgens, each of which may independently influence GLUT4 expression and skeletal muscle glucose metabolism. For instance, FSH promotes GLUT4 translocation via the PI3K/Akt pathway in granulosa cells [57,58], progesterone exhibits divergent effects depending on tissue context [59,60], and androgens may either enhance GLUT4 expression in healthy muscle [61] or exacerbate insulin resistance upon estrogen loss [62,63]. Therefore, changes in GLUT4 in the OVX model may result from the combined effects of multiple hormones. To exclude this confounder, we established an *in vitro* EsD model using phenol red‑free medium and charcoal‑stripped serum, which depletes estrogen without altering other hormones. EsD cells recapitulated the key phenotypes observed in OVX rats—reduced GLUT4 expression, impaired glucose uptake, and mitochondrial dysfunction—and these defects were fully rescued by an ERα agonist. This consistency strongly suggests that estrogen deficiency per se, rather than secondary changes in FSH, progesterone, or androgens, is the primary driver of PFM atrophy and GLUT4 downregulation.

The present study still has some limitations. We have demonstrated that the ERα/GLUT4 pathway-regulated glucose metabolism is related to PFM atrophy caused by estrogen deficiency, but the mechanism of how the disorder of glucose metabolism leads to PFM atrophy is not fully investigated. Moreover, as discussed above, the precise molecular mechanism by which ERα regulates GLUT4 expression remains to be further elucidated.

## 5. Conclusion

In conclusion, our study highlights the estrogen-regulated ERα/GLUT4 pathway as a key regulatory factor in the complex regulatory network of PFM, providing a theoretical basis for the prevention and treatment of PFD and skeletal muscle atrophy in clinical settings.

## Supporting information

S1 File Raw images. (PDF)
