# Supplementary figures and images for: Estrogen deficiency induces pelvic floor muscle atrophy via ERα/GLUT4 pathway
Source: PLoS One. 2026 May 14;21(5):e0349371. doi: 10.1371/journal.pone.0349371 (PMC13175364; doi:10.1371/journal.pone.0349371)

2B

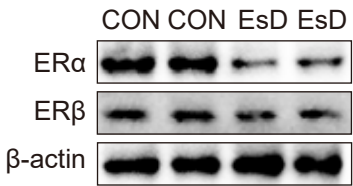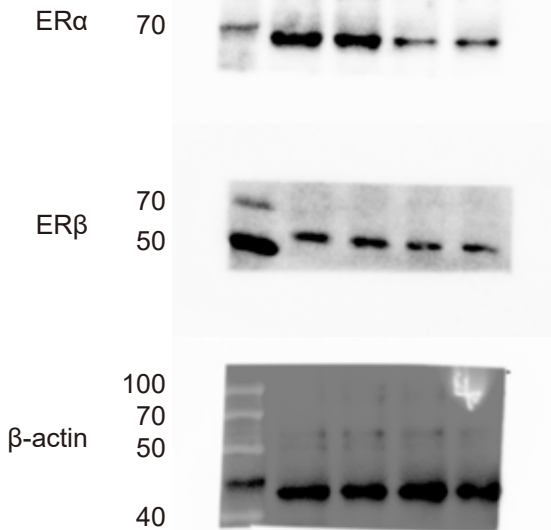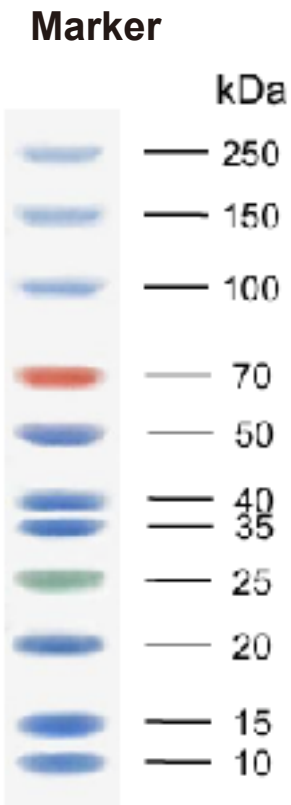

2N

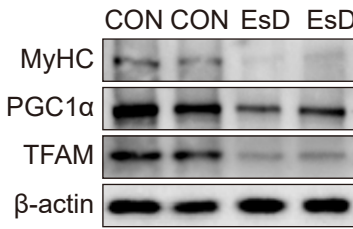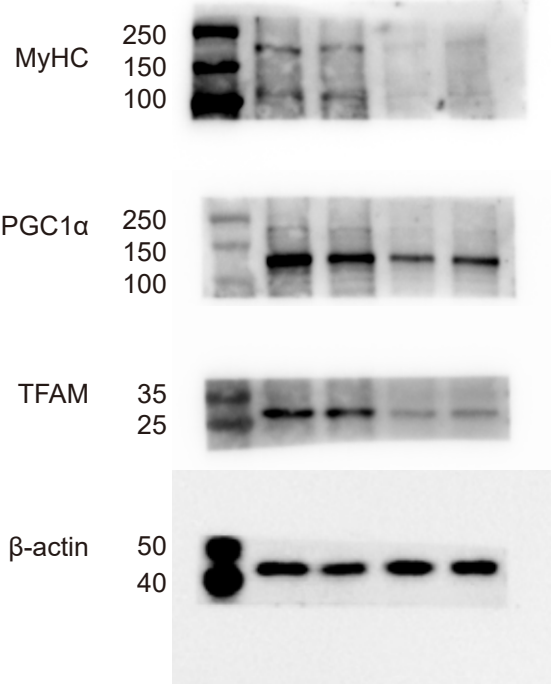

3L

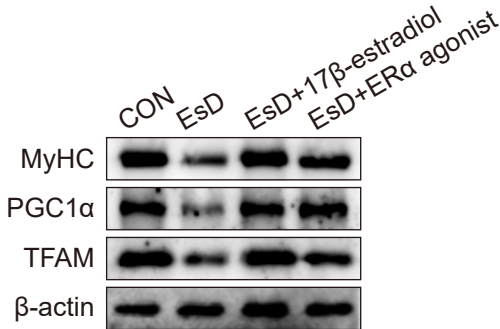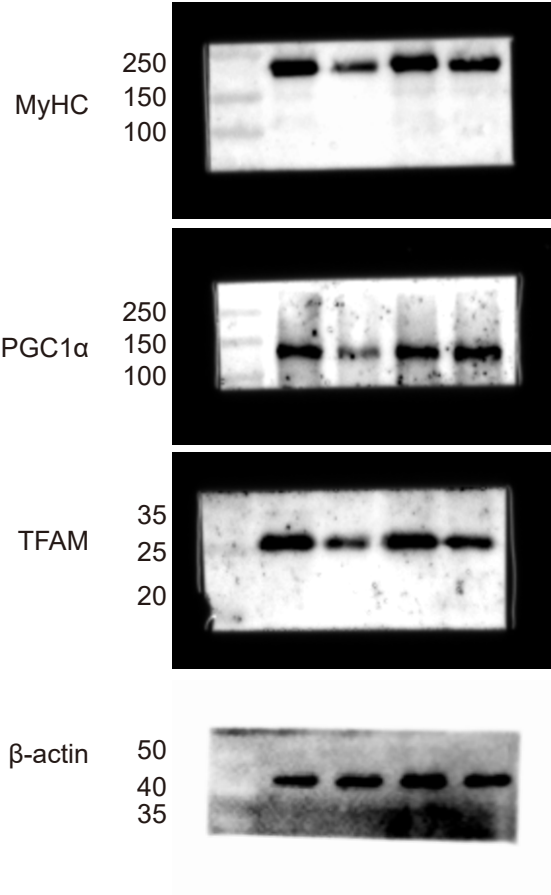

5B

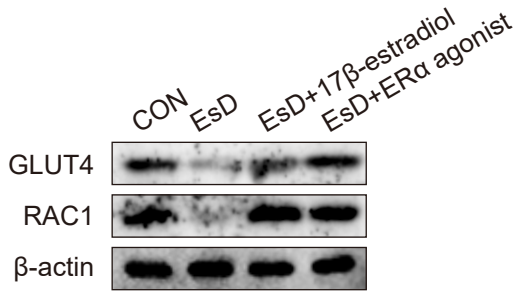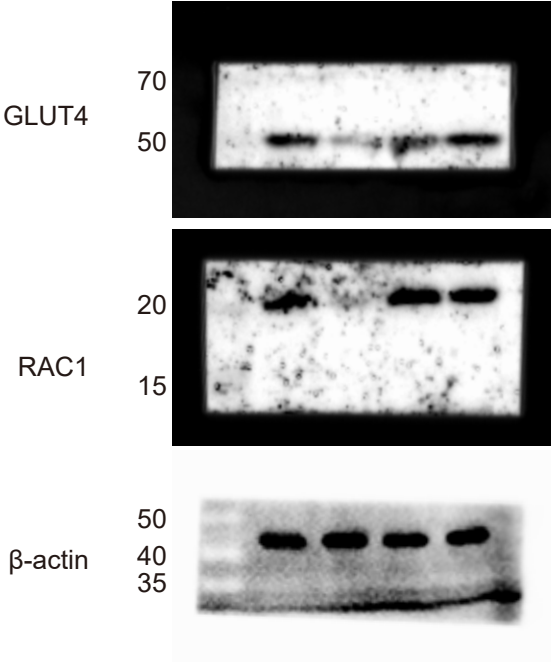

5I

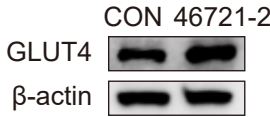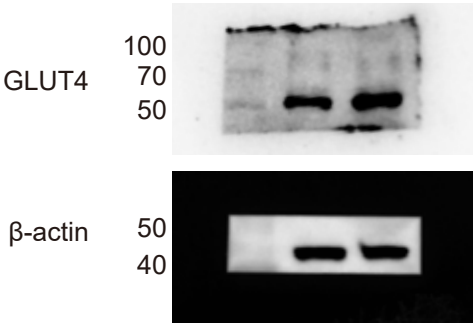

5N

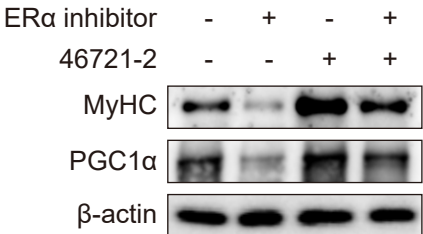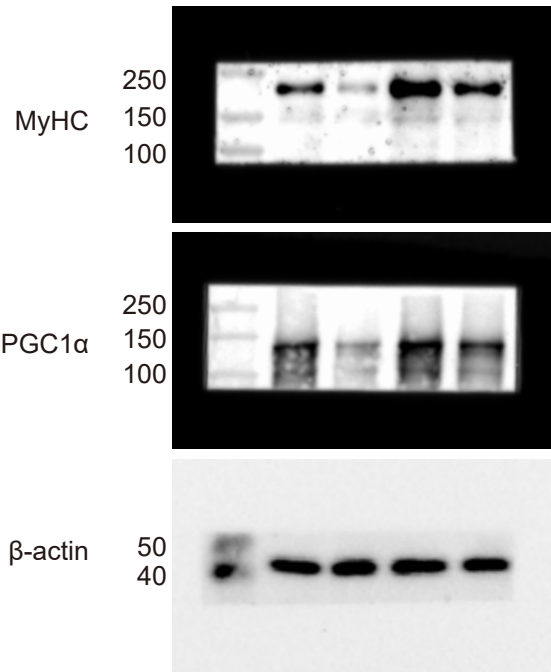

6H

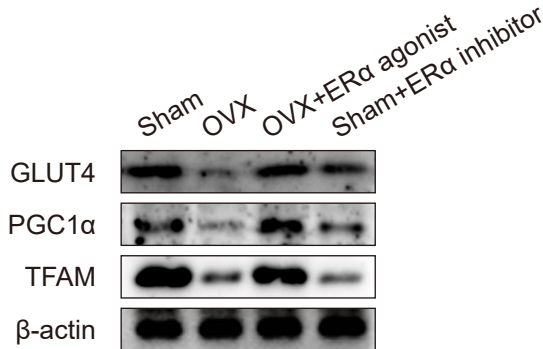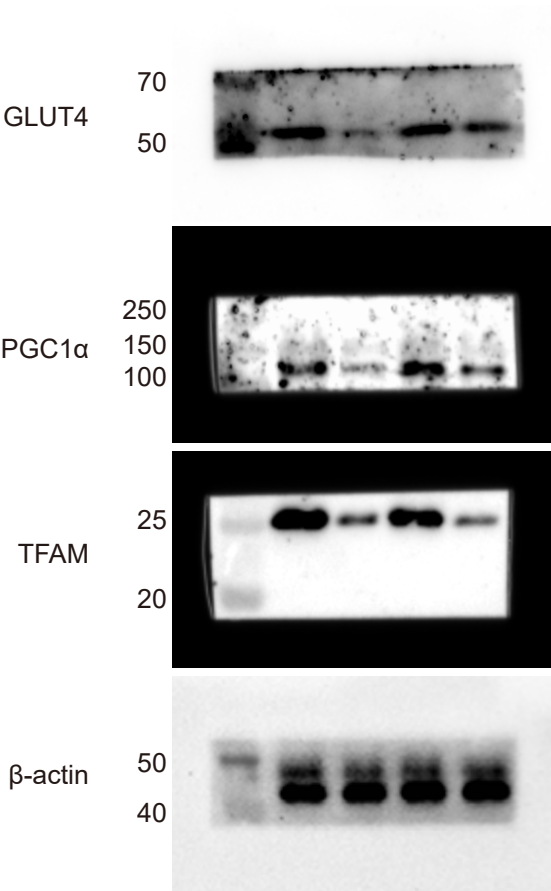

Supplement: S1 File — (PDF) [file pone.0349371.s001.pdf]
